# A review of interventions triggered by hepatitis A infected food-handlers in Canada

**DOI:** 10.1186/1472-6963-6-157

**Published:** 2006-12-08

**Authors:** Andrea C Tricco, Ba' Pham, Bernard Duval, Gaston De Serres, Vladimir Gilca, Linda Vrbova, Andrea Anonychuk, Murray Krahn, David Moher

**Affiliations:** 1 Epidemiology and Biostatistics, GlaxoSmithKline Canada, Mississauga, Ontario, Canada; 2 Chalmers Research Group, Children's Hospital of Eastern Ontario Research Institute, Ottawa, Ontario, Canada; 3 Institute of Population Health, Ottawa, Ontario, Canada; 4 Department of Health Policy, Management and Evaluation, Faculty of Medicine, University of Toronto, Toronto, Ontario, Canada; 5 Centre de Recherche du CHUQ, Universite Laval, Institut national de santé publique du Québec, Canada; 6 University of British Columbia, Department of Health Care and Epidemiology, Vancouver, British Columbia, Canada; 7 Division of Clinical Decision Making, Toronto General Research Institute, University Health Network, Toronto, Ontario, Canada

## Abstract

**Background:**

In countries with low hepatitis A (HA) endemicity, infected food handlers are the source of most reported foodborne outbreaks. In Canada, accessible data repositories of infected food handler incidents are not available. We undertook a systematic review of such incidents to evaluate the extent of viral transmission through food contamination and the scope of post-exposure prophylaxis (PEP) interventions.

**Methods:**

A systematic search of MEDLINE and EMBASE was conducted to identify published reports of incidents in Canada. An expanded search of a news repository (i.e., transcripts from newspapers and newscasts) was also conducted to identify the location and timing of an incident, which was used to retrieve the related report by contacting local public health departments. Data pertaining to case identification, public health risk, PEP interventions, and associated costs was independently abstracted by two reviewers and summarized according to incidents with and without large PEP interventions.

**Results:**

A total of 16 incidents were identified from 1998–2004. There were approximately 3 incidents requiring public notification per year. Only 12.5% of incidents were described in published reports, indicating that published data significantly underestimated the number of incidents and PEP interventions. Data pertaining to the remaining incidents was unpublished, sparse and highly dispersed at the local public health level.

Six of the 16 incidents required large PEP interventions to immunize on average 5000 potentially exposed individuals. Secondary transmission was low. Characteristics of incidents requiring large PEP interventions included potentially infectious food handlers working with uncooked food for a prolonged duration in high-volume grocery stores in high-density urban areas.

**Conclusion:**

Infected food handlers with hepatitis A virus (HAV) requiring public notification are not infrequent in Canada. Published data severely underestimated the burden of PEP intervention. Better and consistent reporting at the local and national level as well as a national data repository should be considered for the management of future incidents.

## Background

In developed countries, foodborne or waterborne hepatitis A (HA) outbreaks are relatively uncommon [1]. However, infected food handlers remain the source of most reported foodborne outbreaks [2]. In many low endemicity countries, the potential for food contamination from an infected food handler is a recognized public health concern [3]. In these countries, a large proportion of the population has never been exposed or vaccinated against hepatitis A virus (HAV) and is thus susceptible to infection during potential outbreaks [4]. In Canada, an infected produce worker in a grocery store triggered a post-exposure prophylaxis (PEP) vaccination campaign of over 19,000 potentially exposed residents, causing substantial disruption to an urban community [5].

There are currently no data repositories for incidents involving infected food handlers, although an early detection system for foodborne outbreaks is in place in Canada [6]. Laboratory-confirmed cases infected with HAV are notifiable on a provincial/territorial and national level, but reporting of follow-up investigations are not mandatory beyond local public health units. Consequently, data are not consolidated and often kept in diverse locations [7]. Further encumbering this issue is the fact that only a small proportion of HAV cases are notified. According to USA data, only 1 in 10 cases are reported via disease notification systems [8]. We undertook a systematic review of infected food handler incidents to evaluate the extent of viral transmission through food contamination and the scope of PEP interventions.

## Methods

A literature search of MEDLINE (1966; year of inception to March 2005) and EMBASE (1980; year of inception to March 2005) was conducted (keywords "hepatitis" and "Canada") to identify published reports of infected food handler incidents in Canada. All searches were conducted using the OVID interface. An expanded search (keywords "hepatitis' AND "food" AND "Canada") of a news repository (FPinfoMart and Newscan; transcripts from over 200 national, provincial and local newspapers and newscast sources: 2000 – the year of establishment – to March 2005) was also conducted to identify the location and timing of an incident. This information was used to retrieve the related unpublished report by contacting the local public health department [9,10]. Archives for the Canada Diseases Weekly Report (CDWR; 1975 – Dec. 1991, the last year of reporting) and Canada Communicable Disease Report (CCDR; 1992 – Mar. 2005) were also consulted [11].

A report was included if it described the incident of an infected food handler in a food establishment in Canada. Citations, news clips (e.g., titles and the first few sentences), full-text news articles (i.e., newspaper and newscast articles), and full-text published reports were reviewed independently by two reviewers. Disagreements were resolved through discussion.

Using the Centers for Disease Control and Prevention framework for the investigation of infectious disease outbreaks [12,13], data pertaining to case identification, public health risk, PEP interventions, and associated costs was independently abstracted by two reviewers. Data was summarized according to the extent of post-exposure prophylactic intervention by the local public health in dealing with an infected food handler. Incidents with limited PEP interventions were those that required 1) a public notification to advise the public and healthcare providers to watch out for potential HAV-related symptoms, and/or 2) immunization of close contacts (e.g., family, colleagues at work). In contrast, incidents with large PEP interventions were those that required 1) a public notification that included eligibility criteria for vaccination and 2) public immunization campaigns.

## Results

### Literature search

**Figure 1:**
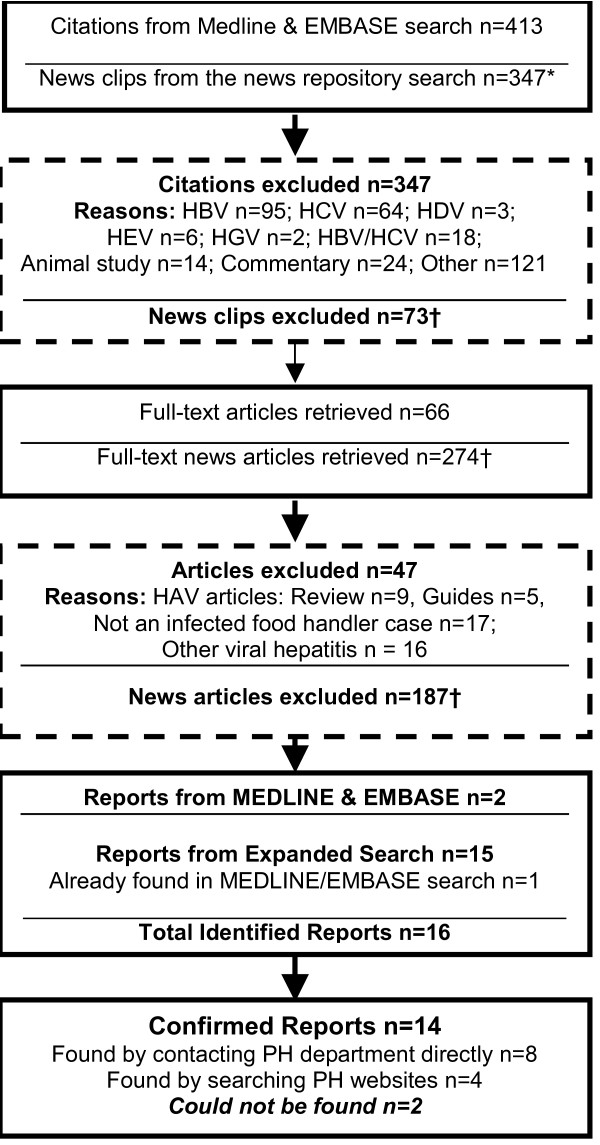
Study flow. Notes: *This number is an approximation due to the commercial coverage of media sources by the news repository. †Reason for exclusion of news clips and news articles were not categorized, as only the location and timing data were used.

Between 1998 and 2004, the systematic review identified 16 incidents of infected food handlers in Canada in total (Table 1). Only two of these (12.5%) were described in published communicable disease reports and identified through MEDLINE (Figure 1) [14,15].

The expanded search identified the location and timing of 15 incidents [5,14-28], one of which was also identified through the MEDLINE search [15]. This search was sensitive to incidents requiring public notification. Subsequently, 3 public health reports and 12 public notifications were obtained (Figure 1, Table 1).

### Case identification

In Canada, approximately 3 incidents of infected food handlers requiring public notification occurred per year between 2001 and 2004 (Table 1). There were no secondary cases reported in 12 of the 16 infected food handler cases, most possibly due to timely intervention, high hygiene standards or non-reporting. Most cases were laboratory confirmed (Table 2). Cause of infection was identified in 3 of the 16 incidents, including infection potentially related to high risk sexual activities [14], contact with visitors from high-endemic countries [15], and food contamination from another infected food handler [17].

The two incidents identified through MEDLINE were described in CCDR reports [14,15]. In the first report, a link analysis found a 6-week gap between case confirmation and public health notification. Several symptomatic HAV cases, all linked to the restaurant where the index case worked, were hospitalized and laboratory-confirmed at the local hospital. Despite these test results, it was a restaurant employee that notified the local public health unit after noticing jaundice symptoms among these cases [14]. In the second report, prompt reporting and quick follow-up of a case from a deli led to the largest immunoglobulin campaign found in this systematic review (n = 5400; Table 1) [15].

### Evidence of public health risk

The 16 incidents occurred in 8 restaurants, 4 grocery stores, 2 hospital food services and 2 food markets (Table 2). Limited PEP interventions were conducted in 7 of the 8 incidents in restaurants. Large PEP interventions were conducted in 3 of the 4 incidents in grocery stores. Incidents requiring a large PEP intervention were associated with 1) the potential for contamination of uncooked food, 2) case notification within 14 days of symptom onset (e.g., within an acceptable window for PEP intervention), 3) prolonged duration of public exposure prior to notification, and 4) relatively large number of potentially exposed clients due to high-volume stores located in high-density urban areas [5,17,20]. The median duration of exposure was 18.5 days and 8 days for incidents with large and limited PEP intervention, respectively. In two incidents involving hospital food facilities, the risk of repeated exposure was balanced by high hygiene practice and the existence of standard procedures for infectious disease control [26,28].

### Post-Exposure Prophylactic interventions

Large PEP interventions typically included communicable disease investigation [5,14,15,20], food inspection and control [5,17], risk communication (e.g., news conference, media releases, media interviews, hot-lines and websites [5,20]), immunization clinics [5,15,17,19,20,23], and surveillance of secondary cases [5]. The median number of immunized individuals was 5,750 (range 550 – 19,208) in incidents with large PEP intervention (Table 2). In the majority of incidents, risk communication included details regarding eligibility criteria for immunization and a qualitative statement regarding the potential risk to the public (Table 2). Food inspection and control was only performed in a few of incidents with limited PEP intervention, yet most of these required risk communication to the public (Table 2).

### Public health cost

Data regarding resource utilization and direct cost to public health was limited, except in the incident with the largest PEP intervention [5]. The total direct cost to public health was $35 per person immunized in a PEP campaign of approximately 19,000 potentially exposed individuals. This included an average CND $20 for the cost of vaccine and $15.17 for vaccine administration (i.e., in lieu of vaccine clinics, inspections, and the hotline).

## Discussion

This systematic review has a number of limitations. The literature search could only identify incidents with a public health notification. These incidents were evaluated to represent an infection risk to those who consumed food prepared by HAV infected food handlers and represent a small and selective sample of all reported incidents of infected food handlers [2]. In other studies, approximately 8% of reported HAV cases involve infected food handlers, approximately 60% of those continue working while potentially infectious, but only 7% represent an infection risk to those who ate food they had prepared [2,12]. Furthermore, the data we collected was sparse; only one out of every four identified incidents was adequately reported. Finally, although the expanded search was based upon a news repository of a large number of data sources, it has only been offering comprehensive coverage since 2000 [9].

Despite these limitations, some major findings could be derived from the consolidated data. Highly publicized infected food handlers were not infrequent in Canada. Only two of the 16 incidents were described in published communicable disease reports, indicating that published data severely underestimated the number of incidents and PEP interventions. These two were probably published due to their atypical nature, as described above. Data pertaining to the remaining incidents was unpublished, sparse and highly dispersed at the local public health level. From a public health perspective, HAV outbreaks and HAV interventions triggered by infected food handlers should be reported and shared nationally, perhaps through the CCDR. Relying on published data only will result in an under-estimation of the burden of public health interventions.

Large PEP interventions to immunize on average 5000 potentially exposed clients were required in six of the 16 incidents. Secondary transmission was low, although this could be due to under-reporting, timely intervention or high hygiene standards. Characteristics of incidents requiring large PEP interventions included potentially infectious food handlers working with uncooked food for a relatively prolonged duration in high-volume grocery stores located in high-density urban areas. Evaluating suspected cases, assessing the need for PEP intervention and implementing necessary control measures are common and time-consuming tasks for public health departments [2]. Factors associated with large PEP interventions identified through this research can facilitate the evaluation as to whether such intervention is merited.

In Canada, there are currently no data repositories for HAV outbreaks or PEP interventions triggered by HAV infected food handlers. Laboratory-confirmed cases infected with HAV are notifiable on a provincial/territorial and national level, but reporting of follow-up investigations are not mandatory beyond local public health units. Only four of the 16 incidents identified were documented with sufficient detail at the local public health level. In one instance, the lack of communication seemed to contribute to secondary cases that were otherwise preventable [14]. Increased surveillance, better communication and data sharing would contribute towards better management of future cases. Members of our group are currently conducting a broad and detailed survey of infected food handlers in Canada.

Reliable data on infected food handlers is required to accurately evaluate the burden of PEP interventions. For example, 4,000 more people were vaccinated in the incident with the largest PEP intervention identified here [5] than the total number of 15,000 vaccinees during one of the largest HAV outbreaks in Canada between 1995 and 1997 [29]. In fact, PEP intervention triggered by a single infected food handler could be more costly to public health than the control of a peak outbreak in recent years. It has been suggested that the cost associated with the management of infected food handlers should be accounted for in cost-effectiveness evaluations of control programs. Currently, these management costs are not commonly accounted for in such analyses [30].

A previous cost effectiveness analysis suggested that vaccinating food service workers in states with elevated HAV rates prior to routine childhood vaccination was cost-effective [31]. However, a simulation study concluded that vaccinating restaurant employees was unlikely to be economical from either the restaurant owner or the societal perspective, even during HAV epidemics [32]. Our results showed that limited PEP interventions were sufficient to contain potential transmission from infected food workers in restaurants. Large PEP interventions might, however, become necessary in infected cases working in high-volume food establishments in high-density urban areas.

Countries such as Canada with low HA endemicity have experienced declining incidence of new cases of HAV in the past decade [33]. However, this has led to a decreased prevalence of antibody to HA in the population, resulting in an adult population not protected against HAV [33]. In the meantime, sporadic outbreaks of foodborne HA, related and unrelated to a food handler, continue to occur [2,33-35]. Recently, contaminated green onions were served to customers of a single restaurant in Pennsylvania, leading to a large outbreak in the United States [34].

HAV-infected food handlers have been the source of most reported foodborne HA outbreaks. Six outbreaks that occurred in the 1990s have recently been document in a review of HA foodborne transmission [2]. Our results are consistent with observations elsewhere; a single infected food handler can transmit HAV to dozens or even hundreds of individuals and cause substantial economic burden [2,30]. Specific public health interventions [34,36,37] are required to contain this form of transmission until high levels of immunity are achieved across all age groups, perhaps as a result of routine HA vaccination [33,35].

Universal immunization of young children, implemented in some western and south-western parts of the United States, substantially reduced the incidence of HAV [38]. Due to its cost-effectiveness potential, this policy is being considered for other regions [39,40], including calls for its expansion nationally [41]. In Canada, the current immunization strategy to control HA is to vaccinate groups at risk [42]. However, this strategy has been shown as ineffective among travelers [43] and very limited data are available on its effectiveness in other risk groups. Recent seroprevalence studies indicate that only 3% of children ages 8–13 are protected against HAV [44], whereas disease acquisition occurs in adulthood with approximately 10% of Canadians infected by ages 24–29 [11,45-47]. Concern with this seemingly lack of protection has led to calls for the reassessment of the current policy regarding HA vaccination [4,44,48]. Universal vaccination could eliminate the spectrum of PEP interventions related to HA cases in food handlers that emerge periodically [33].

The importance of foodborne viral infections is increasingly recognized [2,3]. Food handlers can transmit infection during preparation or serving; fruit and vegetables may be contaminated by fecally contaminated water used for growing and washing. The globalization of the food industry and the ease of cross-border shipment of fresh and frozen food means that a contaminated food item may not be limited to one location. To meaningfully monitor increases or decreases in foodborne disease requires an effective surveillance system at the local and national levels. This should include standardized reporting of foodborne incidents and a national repository for consolidated data on these incidents.

## Conclusion

Infected food handlers with HAV requiring public notification are not infrequent in Canada. Published data severely underestimated the burden of PEP intervention. Better and consistent reporting at the local and national level as well as a national data repository should be considered for the management of future incidents.

## Competing interests

Funding for this systematic review was provided by GlaxoSmithKline Canada. ACT has held various research contracts with the R&D department of GlaxoSmithKline, Canada. BP is employed by GlaxoSmithKline, Canada. AA has held various research contracts with the R&D department of GlaxoSmithKline, Canada. BD, GDS, and VG have received research funding from the R&D department of GlaxoSmithKline, Canada. LV, MK, and DM declare that they have no competing interests.

## Authors' contributions

ACT contributed to the development of the research question and methodology, conducted the literature searches, article screening, data abstraction, project management, and interpretation of the results. BP contributed to the development of the research question and methodology, article screening, data abstraction, project management, and interpretation of the results. BD, GDS and VG contributed to the development of the research question and methodology, provided data from a survey of infected food handlers reported to Quebec public health units (data not shown but used in the interpretation of the current results), provided guidance on obtaining public health reports, and interpretation of the results. LV contributed to the development of the methodology, provided guidance on obtaining public health reports, interpretation of the results, and is currently conducting the follow-up national survey. AA contributed to data abstraction, project management, and interpretation of the results. MK contributed to the development of the research question and methodology, obtained public health reports, and interpretations of the results. DM contributed to the development of the research question and methodology, provided guidance on handling of grey literature in a systematic review, and interpretation of the results. All of us contributed to the manuscript writing and approved the final version of the manuscript.

## Pre-publication history

The pre-publication history for this paper can be accessed here:



## Figures and Tables

**Table 1:** Identified incidents of HA infected food handlers with confirmed public health reports (n = 16, 1998–2004)

**Timing of discovery**	**City, province**	**Index case, location**	Total # cases*	**Public health Intervention**	**# immunized**	**Search method‡, reference #**
Sep 2004	Okanagan, BC	bar worker, restaurant	1	vaccine	NR	expanded, [27]
Apr 2004	Port Clements, BC	vegetable department worker, food market	1	vaccine	NR	expanded, [26]
Apr 2004	Burlington, ON	food service worker, hospital cafeteria	1	doctor referral†	NA	expanded, [25]
Dec 2003	Burlington, ON	food service worker, restaurant	1	doctor referral†	NA	expanded, [24]
Jul 2003	Grand Prairie, AB	part-time employee, restaurant	1	IG	>550	expanded, [23]
Apr 2003	Whistler, BC	food service worker, 2 restaurants	1	doctor referral†	NA	expanded, [22]
Apr 2003	Winnipeg, MB	cases were linked to a restaurant	17	doctor referral†	NA	expanded, [21]
Sep 2002	London, ON	food handler in produce department, grocery store	1	vaccine	16,320	expanded, [20]
Sep 2002	Edmonton, AB	food handler, ethnic specialty food market	1	IG	2,300	expanded, [19]
Aug 2002	Toronto, ON	food handler in produce department, grocery store	3	vaccine & IG	19,208	expanded, [5]
Mar 2002	Vancouver, BC	food service worker, 3 grocery stores	8	vaccine & IG	6,100	expanded, [17]
Apr 2002	Vancouver, BC	server, restaurant	1	doctor referral†	NA	expanded, [28]
Mar 2002	Edmonton, AB	worker, lounge	1	IG	50	expanded, [18]
Apr 2001	Victoria, BC	food handler, hospital food services	1	vaccine	NR	expanded, [16]
Jan 2001	Edmonton, AB	deli worker, grocery store	1	IG	5,400	MEDLINE & expanded [15]
Nov 1998	Montérégie, QB	owner and chef, restaurant	7	IG	NR	MEDLINE [14]

Abbreviations: HA hepatitis A, AB Alberta, BC British Columbia, ON Ontario, QB Quebec, MB Manitoba, NR not reported, NA not applicable, IG administration of hepatitis A immune serum globulin, PEP post-exposure prophylaxis.

Notes: *Reported cases only. †Public health asked individuals who displayed any symptoms of hepatitis A to get tested through a family physician. ‡Search method refers to Medline/EMBASE search or the expanded search (i.e., searching news repository for timing and location, which was then verified by the respective public health report).

**Table 2:** Public health investigation of infected food handlers (n = 16, 1998–2004)

**Items**	**Large PEP intervention (n = 6)**	**Limited PEP intervention (n = 10)**
**Case identification**		
*Case confirmation and cause of infection*		
Laboratory confirmed case	6	7
Known cause of infection	2	1
*Duties*		
Food/drink worker in restaurant/lounge	1	7
Food handler in produce/deli section, grocery/food store	4	0
Food worker in hospital food services/cafeteria	0	2
Food/veggie worker in food market	1	1
*Hygiene practice*		
High standard/met requirements for food handling	4	6
		
**Evidence of public health risk**		
*Timing of discovery*		
Within 14 days of symptom onset	5	4
> 14 days of symptom onset	0	6
Estimated duration of public exposure in days*		
Median (range) [# cases with data]	18.5 (4, 23) [n = 6]	8 (3, 45) [n = 9]
*Qualitative estimate of the number of exposed individuals*		
Estimated number in hundreds or thousands	5	1
Unspecified number of clients over an exposed duration	1	9
		
**Post-exposure prophylaxis intervention**		
*Immunization*		
Vaccine (or vaccine and immunoglobulin)	3	2
Immunoglobulin	3	4
Doctor referral	0	4
*Number of immunized individuals*†	5750 (550, 19208) [n = 6]	All contacts [n = 2], 50 [n = 1]
*Food safety inspection and control*		
Recall, remove & clean up potentially contaminated food	3	1
*Risk communication*		
Eligibility criteria for vaccination	6	8
Communicate estimated risk to the public	6	9
		
**Public health cost**		
Reported resources utilization and/or direct cost	5	2

Abbreviations: PEP: post-exposure prophylaxis.

Notes: *Estimated duration of exposure as specified in eligibility criteria for immunization, or number of days working while potentially infectious. †Median (range) [# cases with data] for large public health interventions and category (i.e., IG all contacts, IG 50 close contacts) [# cases with data] for limited public health interventions.

## References

[B1] Acheson DW, Fiore AE (2004). Preventing foodborne disease--what clinicians can do. N Engl J Med.

[B2] Fiore AE (2004). Hepatitis A transmitted by food. Clin Infect Dis.

[B3] Koopmans M, Vennema H, Heersma H, van SE, van DY, Brown D, Reacher M, Lopman B (2003). Early identification of common-source foodborne virus outbreaks in Europe. Emerg Infect Dis.

[B4] Scheifele DW (2005). Hepatitis A vaccines: the growing case for universal immunisation of children. Expert Opin Pharmacother.

[B5] Basrur V (2002). Toronto Staff Report: Hepatitis A at a Toronto grocery store. Toronto Public Health.

[B6] (2004). Global Public Health Intelligence Network.

[B7] Schuster CJ, Ellis AG, Robertson WJ, Charron DF, Aramini JJ, Marshall BJ, Medeiros DT (2005). Infectious disease outbreaks related to drinking water in Canada, 1974-2001. Can J Public Health.

[B8] Armstrong GL, Bell BP (2002). Hepatitis A virus infections in the United States: model-based estimates and implications for childhood immunization. Pediatrics.

[B9] (2005). FPInfomart Website. http://www.fpinfomart.ca/.

[B10] (2005). Newscan Website. www.newscan.com.

[B11] Pham B, Duval B, De SG, Gilca V, Tricco AC, Ochnio J, Scheifele DW (2005). Seroprevalence of Hepatitis A Infection in A Low Endemicity Country: A Systematic Review. BMC Infect Dis.

[B12] (2003). Foodborne transmission of hepatitis A--Massachusetts, 2001. MMWR Morb Mortal Wkly Rep.

[B13] Reingold AL (1998). Outbreak investigations--a perspective. Emerg Infect Dis.

[B14] Fortin A (1998). Hepatitis A in restaurant clientele and staff--Quebec. Can Commun Dis Rep.

[B15] Honish L (2001). Hepatitis A infected food handler at an Edmonton, Alberta retail food facility: public health protection strategies. Can Commun Dis Rep.

[B16] Purdy C (2001). Hospital food worker diagnosed with hepatitis. The Edmonton Journal.

[B17] Authority VCH (2002). Hepatitis A in a Capers grocery store in Vancouver. Infectious Diseases News Brief.

[B18] Honish L (2002). Hepatitis A in an Edmonton lounge establishment.

[B19] Honish L (2002). Hepatitis A in an Edmonton ethnic speciality food market.

[B20] Pollett GL (2002). Middlesex-London Health Unit Report No. 128-02: Hepatitis A in a foodhandler.

[B21] Authority WRH (2003). Hepatitis A in a popular family restaurant in Winnipeg: Tuxedo Village Restaurant.

[B22] O'Conner B, Spitz A (2003). Hepatitis A alert - Umberto's Restaurant - Whistler.

[B23] Health. PC (2003). Update hepatitis A case in Grande Prairie.

[B24] Department HRH (2004). The regional municipality of Halton: Hepatitis A advisory.

[B25] Department HRH (2004). Media release: Hepatitis A advisory.

[B26] Authority NH (2004). Port Clements Health Advisory: hepatitis A case confirmed.

[B27] Control BCCD (2004). Interior health issues alert for patrons of Kelowna M.Y. Martini Place and Lounge Restaurant.

[B28] (2002). Hepatitis prompts vaccination plan. Canadian Press.

[B29] Allard R, Beauchemin J, Bedard L, Dion R, Tremblay M, Carsley J (2001). Hepatitis A vaccination during an outbreak among gay men in Montreal, Canada, 1995-1997. J Epidemiol Community Health.

[B30] Dalton CB, Haddix A, Hoffman RE, Mast EE (1996). The cost of a food-borne outbreak of hepatitis A in Denver, Colo. Arch Intern Med.

[B31] Jacobs RJ, Grover SF, Meyerhoff AS, Paivana TA (2000). Cost effectiveness of vaccinating food service workers against hepatitis A infection. J Food Prot.

[B32] Meltzer MI, Shapiro CN, Mast EE, Arcari C (2001). The economics of vaccinating restaurant workers against hepatitis A. Vaccine.

[B33] Di GL, Dienstag JL (2005). Hepatitis A--the price of progress. N Engl J Med.

[B34] Wheeler C, Vogt TM, Armstrong GL, Vaughan G, Weltman A, Nainan OV, Dato V, Xia G, Waller K, Amon J, Lee TM, Highbaugh-Battle A, Hembree C, Evenson S, Ruta MA, Williams IT, Fiore AE, Bell BP (2005). An outbreak of hepatitis A associated with green onions. N Engl J Med.

[B35] Hutin YJ, Pool V, Cramer EH, Nainan OV, Weth J, Williams IT, Goldstein ST, Gensheimer KF, Bell BP, Shapiro CN, Alter MJ, Margolis HS (1999). A multistate, foodborne outbreak of hepatitis A. National Hepatitis A Investigation Team. N Engl J Med.

[B36] Amon JJ, Devasia R, Xia G, Nainan OV, Hall S, Lawson B, Wolthuis JS, Macdonald PD, Shepard CW, Williams IT, Armstrong GL, Gabel JA, Erwin P, Sheeler L, Kuhnert W, Patel P, Vaughan G, Weltman A, Craig AS, Bell BP, Fiore A (2005). Molecular epidemiology of foodborne hepatitis a outbreaks in the United States, 2003. J Infect Dis.

[B37] Koopmans M, Duizer E (2004). Foodborne viruses: an emerging problem. Int J Food Microbiol.

[B38] Jenson HB (2004). The changing picture of hepatitis A in the United States. Curr Opin Pediatr.

[B39] Rosenthal P (2003). Cost-effectiveness of hepatitis A vaccination in children, adolescents, and adults. Hepatology.

[B40] Jacobs RJ, Greenberg DP, Koff RS, Saab S, Meyerhoff AS (2003). Regional variation in the cost effectiveness of childhood hepatitis A immunization. Pediatr Infect Dis J.

[B41] Jacobs RJ, Meyerhoff AS, Zink T (2005). Hepatitis A immunization strategies: universal versus targeted approaches. Clin Pediatr (Phila).

[B42] Immunization NAC (2002). Canadian Immunization Guide.

[B43] De Serres G, Duval B, Shadmani R, Boulianne N, Pohani G, Naus M, Douville FM, Rochette L, Ward BJ, Kain KC (2002). Ineffectiveness of the current strategy to prevent hepatitis A in travelers. J Travel Med.

[B44] Duval B, De Serres G, Gilca V, Ochnio J, Scheifele DW (2005). Nationwide Canadian study of hepatitis A antibody prevalence among children eight to thirteen years old. Pediatr Infect Dis J.

[B45] Ochnio JJ, Patrick D, Ho M, Talling DN, Dobson SR (2001). Past infection with hepatitis A virus among Vancouver street youth, injection drug users and men who have sex with men: implications for vaccination programs. CMAJ.

[B46] Ochnio J, Scheifele DW, Lightle G, Ho M (1997). Hepatitis A is uncommon among children in Vancouver: results of an antibody prevalence survey in grade six students. BC Health Dis Surveillance.

[B47] Moses S, Mestery K, Kaita KDE, Minuk GY (2002). Viral hepatitis in a Canadian street-involved population. Can J Public Health.

[B48] Scheifele DW, Ochnio J (2002). Hepatitis A vaccine: is it being used to best advantage?. CMAJ.

